# Exposure to Secondhand Tobacco Smoke at Airport Terminals

**DOI:** 10.1155/2019/9648761

**Published:** 2019-02-03

**Authors:** Michael Zhang, Alejandro D. Garcia, Maritere Zamora, Isabella A. Anderson, David F. Jativa

**Affiliations:** ^1^Department of Medicine, Cleveland VA Hospital, Cleveland, OH 44106, USA; ^2^School of Health Sciences, Miami Dade College, Miami, FL 33132, USA; ^3^Baptist Health, Florida International University, Miami, FL 33199, USA; ^4^Department of Medicine, Florida Atlantic University, Boca Raton, FL 33431, USA; ^5^Department of Medicine, Aventura Hospital and Medical Center, Aventura, FL 33180, USA

## Abstract

**Background:**

Airports may represent significant sources of secondhand smoke (SHS) exposure for both travelers and employees. While previously common smoking rooms have largely disappeared from US airports, smoking continues to occur outdoors at terminal entrances. SHS may be especially high at arrival areas, since they oftentimes are partially enclosed by overhead departures, creating stagnant microenvironments. This study assessed particulate matter <2.5 microns in diameter (PM2.5), a common surrogate for SHS, at airport terminal locations to evaluate both outdoor exposure risk and possible indoor drift of SHS from outdoor sources.

**Methods:**

A convenience sample of nine airport terminal arrival areas in the US state of Florida was surveyed between February and July 2018. PM2.5 levels were assessed outdoors and indoors at terminal entrances and at control areas far into terminal interiors. We also examined the impact of smoking location on SHS exposure by correlating cigarette and passing vehicle counts with PM2.5 levels at terminals with contrasting proximity of designated smoking locations to terminal entrances.

**Results:**

Although outdoor PM2.5 levels (mean 17.9, SD 6.1 *µ*g/m^3^) were significantly higher than indoors (*p* < 0.001), there was no difference between indoor areas directly inside terminal entrances and areas much further interior (mean 8.8, SD 2.6 vs mean 8.5, SD 3.0 *µ*g/m^3^, *p*=0.49). However, when smoking areas were in close proximity to terminal entrances, the number of lit cigarettes and vehicular traffic per minute predicted 70% of the variance of PM2.5 levels (*p* < 0.001), which was attributable mostly to the cigarette number (*β* = 0.83; 95% CI (0.55 to 1.11); *p* < 0.001). This effect was not observed at smoking areas further away.

**Conclusion:**

PM2.5 data did not suggest indoor drift from outside smoking. Nevertheless, absolute exposure outdoors was high and correlated with the location of designated smoking areas. Further studies are needed to examine the effect of microclimate formation on exposure risk.

## 1. Introduction

Exposure to secondhand tobacco smoke (SHS), also called passive smoking, has been recognized for decades as a significant public health hazard that confronts healthcare providers and lawmakers alike [1]. Composed of over 7000 chemicals [2], over 250 of which are known toxins and carcinogens, even brief exposure can lead to negative effects [3]. Prolonged exposure to SHS has been linked to numerous diseases, including COPD, cardiovascular disease, and stroke [4], and is believed to be responsible for 9–13% of cancer cases in the nonsmoking population [3, 5]. Overall, the global burden of disease from SHS exposure is estimated to be responsible for some 600,000 deaths and billions of dollars of healthcare costs on a yearly basis [6].

Previous research on SHS in the airport setting has largely focused on exposure to nonsmokers outside of designated indoor smoking lounges. One study in 2010 found that particulate matter less than 2.5 microns in diameter (PM2.5), a common measure of SHS, leaked away from enclosed airport smoking venues and entered the general air circulation, exposing workers and the public to SHS [7]. Another study by the Centers for Disease Control and Prevention (CDC) in 2012 in major US airports with and without smoking lounges found that PM2.5 levels were fourfold greater in nonsmoking areas adjacent to smoking venues. These levels numerically were also higher in public seating areas at randomly selected terminal gates in smoking airports, although this did not reach statistical significance. They agreed that “restriction of smoking to designated areas is not effective in eliminating SHS exposure” [8].

Due to increasing awareness of these hazards, four of the seven offending airports identified by the CDC have since banned indoor smoking altogether [9, 10]. However, studies in various settings have concluded that smoking activity often moves outdoors following the enactment of indoor bans [11], resulting not only in outdoor SHS but also in indoor drift [12, 13]. Anecdotal reports suggest that airports are no exception [14]. Outdoor smoking at airports generally occurs outside of terminals [10], and one report found measureable air nicotine at such locations [15]. These same areas serve as entry and exit points to and from the airport, usually via ground transportation. If smoking activity indeed has moved from indoor lounges to arrival and departure areas outside of airport terminals, travelers and workers alike would be expected to be exposed to outdoor SHS and possibly indoor drift. The risk may be especially pronounced at arrival areas, since they are oftentimes located at the ground level directly beneath departures. These partially enclosed environments can be considered microclimates where particles from both SHS and vehicular exhaust can stagnate in the absence of full air circulation [16], and previous transportation studies in the setting of confined subway stations have also suggested this effect [17, 18].

In the present study, we investigated indoor levels of PM2.5 at multiple airport terminals just beyond terminal entrance and exit points and compared them to levels far into the terminal interiors. We hypothesized that there would be a difference in levels between these locations caused by indoor drift from outdoor pollutants. We also investigated the impact of smoking location on PM2.5 measurements and hypothesized that the placement of designated smoking venues further away from terminal entrances would result in lower levels despite any possible microclimate effects.

## 2. Materials and Methods

### 2.1. Airports

A convenience sample of three public airports in the US state of Florida was selected for this cross-sectional observational study which took place between February and July 2018. Each terminal was sampled separately for a total of nine terminals amongst these airports. The three airports are all considered by the US Federal Aviation Administration (FAA) as “large hub airports,” meaning that each is responsible for at least 1% of annual passenger boardings across the United States [19]. While smoking was prohibited indoors, every airport had designated smoking spaces directly outside terminals in the arrival areas. Like the foregoing CDC study, all data from these airports were de-identified under results and terminals were reported as airports A–C and terminals 1–9. Ethical approval was not required for this study as no measurements were made on human subjects.

### 2.2. Study Protocol

This study was divided into two phases. During the first phase, we aimed to assess the presence of indoor drift of SHS by measuring PM2.5 levels at three separate locations at each terminal: (1) outdoors on the sidewalk in the arrival areas directly outside of terminals; (2) indoors inside terminals within nine meters from the nearest entrance closest to a designated outdoor smoking area; and (3) control areas consisting of randomly selected seating areas well into the terminal interiors. The use of PM2.5 as a surrogate for SHS has been validated in numerous studies [20]. The nine-meter distance was selected based on a prior study which reported that outdoor PM2.5 is capable of drifting at least that distance away from a simulated smoking source [21]. Each area was surveyed for at least 30 minutes to obtain a mean PM2.5 over that interval. The number of lit cigarettes seen while surveying area (1) was also noted. For the second phase, we aimed to assess the impact of smoking location on PM2.5 measurements. We selected two terminals at two different airports: (1) one terminal featuring designated smoking areas that were within nine meters of terminal entrances and (2) another terminal where the closest designated smoking areas were in secluded venues well beyond 20 meters away from terminal entrances. For both terminals, we measured PM2.5 values within a 9-meter radius from the nearest entrance that was closest to a designated outdoor smoking area; this was also where the majority of pedestrian traffic passed through on entry and exit. We surveyed each area for 10 minutes on 20 separate occasions with no more than three surveys per day at any given terminal. We also counted both the number of lit cigarettes seen while surveying, with the presence of at least one lit cigarette as the prerequisite to survey commencement, as well as the number of vehicles passing through a fixed point at the terminal road. For both phases, all fieldwork was conducted between the hours of 1500 and 2200 by two surveyors.

### 2.3. Monitoring Equipment

We used a SidePak AM510 Personal Aerosol Monitor (TSI Inc, St Paul, MI) placed in a backpack with the sampling tube exposed to measure air particulate matter less than 2.5 microns in diameter, which account for almost all respirable suspended particulates from cigarette smoke [22]. This device is a laser photometer that draws in ambient air with a sampling pump and uses light scattering to determine particle mass concentrations, and its use has been extensively validated in earlier studies on SHS [20]. Based on previous research, we used a calibration factor of 0.29 [23], which has been standard for SHS across multiple studies [24]. Based on an earlier precedent, no calibration changes were made for traffic emissions [25]. Per manufacturer's instructions, the device was zero-calibrated with a HEPA filter before each use and the flow rate set to 1.7 l/min. One-minute or 10-second logging intervals were used depending on the study phase (see the following section), and all data were exported immediately to a laptop. A generic hand tally clicker was used to count passing vehicles.

### 2.4. Statistical Analysis

For phase I, survey means for each of the nine terminals studied at each of the three specified locations were provided for a total of 27 data points. These PM2.5 means were grouped into the three locations as described in the study protocol and compared using the nonparametric Kruskal–Wallis one-way analysis of variance (ANOVA) test. Statistical significance, set at *α* = 0.05, was further assessed with post hoc Dunn's testing. For phase II, 10-second PM2.5 data over each individual survey lasting 10 minutes were averaged to provide an arithmetic mean for a total of 20 data points. Multiple linear regression analysis was performed to observe the impact of covariates (cigarette and passing vehicle number per minute) on PM2.5 levels in each of the two groups described under the study protocol. 95% confidence intervals (CI) and standardized beta coefficients (*β*) are provided where appropriate. Nonparametric Mann–Whitney *U* (MW) testing was performed on covariates between these groups. All statistical analysis was conducted using JASP version 0.9.1.0, an open source software package (https://jasp-stats.org/).

## 3. Results

### 3.1. Airport Characteristics

Each airport was initially visited and qualitatively surveyed prior to study commencement (Table 1). All airports had outdoor smoking restrictions in place with designated areas provided for smokers. At two airports, A and C, such areas were located along the pedestrian sidewalk directly outside of the terminal entrances, although airport C restricted these areas to the very ends of the terminals only. At airport B, smoking was prohibited along the sidewalk and permitted only in secluded areas across the road over 20 m away from the terminal doors. There were nine terminals total amongst all of these airports.

Architecturally, all airports feature arrival areas located beneath and partially enclosed by departure areas overhead, potentially creating microclimates that limit air circulation.

### 3.2. Assessment of Indoor Drift of Secondhand Smoke

**Figure 1:**
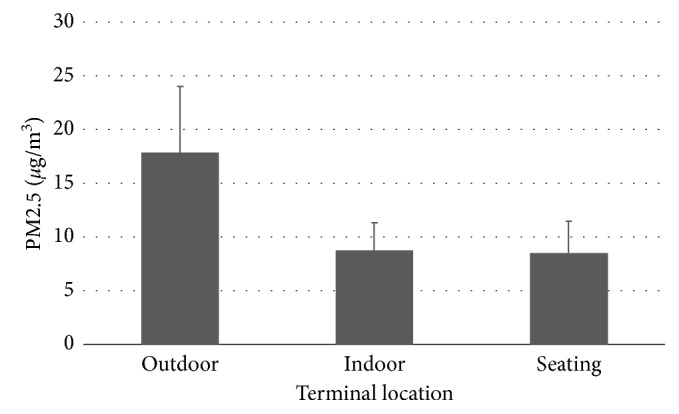
Histogram of PM2.5 measurements by terminal location. The error bars represent the standard deviation of nine measurements at each location.

For phase I of our quantitative study, we visited all nine terminals within these airports and collected PM2.5 data at three locations in each terminal: (1) outdoors in the arrival areas directly outside of terminals; (2) indoors inside terminals within 9 meters from the nearest entrance that was closest to a designated outdoor smoking area; and (3) control areas consisting of randomly selected seating areas well into the terminal interiors (Table 2 and Figure 1).

Kruskal–Wallis testing demonstrated a significant difference in PM2.5 levels amongst these groups (*p* < 0.001) which was due to high outdoor levels; however, there was no difference noted between the indoor setting within the terminal entrances nine meters away from the terminal entrances and seating areas deep in the terminal interiors (*p*=0.49).

### 3.3. Assessment of the Effect of Smoking Location on PM2.5

For phase II, we investigated the impact of smoking location on PM2.5 measurements at airport arrival areas. We selected two contrasting terminals, labeled as terminals 4 and 5, located at different airports. Terminal 4 featured outdoor smoking areas along the sidewalk almost directly outside and well within 9 meters of its entrances. Numerous cigarette butts were noted on the ground even in nonsmoking areas, the presence of which is known to be associated with heavy metal leaching [26, 27]. In comparison, the designated smoking venues of terminal 5 were located past the sidewalk on the other side of the terminal road in secluded terraces well beyond 20 meters away from the terminal entrances. Cigarette number, passing vehicle number, and PM2.5 levels were measured at both terminals within a 9-meter radius from the nearest entrance that was closest to a designated outdoor smoking area (Supplemental Materials S1).

Multiple linear regression analysis showed that altogether, the number of lit cigarettes and vehicular traffic per minute predicted 70% of the variance of PM2.5 levels at terminal 4 (*p* < 0.001). However, this was attributable mostly to the former covariate of lit cigarettes per minute (*β* = 0.83; 95% CI (0.55 to 1.11); *p* < 0.001) instead of vehicles counted per minute (*β* = 0.20; 95% CI (−0.08 to 0.49); *p*=0.15). For terminal 5, these covariates predicted only 26% of the variance of PM2.5 levels (*p*=0.08), with neither reaching statistical significance (*p*=0.1 for both) (Table 3).

Mann–Whitney *U* testing was performed for both covariates as well as PM2.5 levels between the two terminals; PM2.5 was significantly higher in terminal 4 (*p*=0.002, 1-tailed MW) but covariates did not differ (*p*=0.22 for cigarettes/min and 0.26 for vehicles/min, 2-tailed MW).

## 4. Discussion

In this cross-sectional observational study of nine airport terminals at three airports designated by the FAA as “large hub airports,” we found that levels of particulate matter <2.5 microns in diameter (PM2.5) just within the entrances of these terminals did not significantly differ from randomly selected seating areas far into the terminal interiors. This was despite higher PM2.5 levels measured just outside of these terminals, suggesting that any possible indoor drift was negligible. However, we did find that outdoor PM2.5 levels at arrival areas varied significantly depending on the location of designated smoking venues; locations closer to arrival areas were associated with a higher absolute PM2.5 exposure which was attributable largely to cigarette smoke rather than vehicular emissions, while locations further away from arrival areas resulted in lower PM2.5 exposure which was attributable to neither covariate.

Our study is the first in the literature to investigate indoor drift from outside smoking sources in the airport setting and speculate on the peculiar risk in partially enclosed arrival areas. Previous studies on indoor drift from indoor smoking lounges have yielded contrasting results. Lee et al. suggested that leakage from indoor smoking lounges did indeed drift to surrounding areas, placing workers, and the public at risk [7]; however, the lack of descriptive statistics therein renders their report difficult to interpret. In comparison, the study conducted by the CDC did not find any significant difference between indoor PM2.5 levels at airports with indoor smoking lounges and those without [8]. However, that study also lacked the statistical power to produce a meaningful discrimination; although there was a numerical PM2.5 difference between the two types of airports, 11.5 and 8.0 *µ*g/m^3^, respectively, a sample size of around 180 airports would have been required instead of nine to detect a difference between the two groups, assuming the same means. Because only a few airports across the United States still feature indoor smoking lounges, satisfying this figure would have been impossible. Nevertheless, in the current study, it probably would have made little difference had the sample size been greater. The airport that featured the closest smoking areas from its entrances also had two sets of doors separating the terminal interiors from the outside environment. Further, ventilation grating was present not only around the perimeter but also within the space bound by the double doors, with audible air flow at all times. Meanwhile, the other two airports permitted smoking only at a distance from the terminal entrances; one had smoking areas only at the very ends of the terminals while another placed them in secluded areas further down the road. All of these factors probably acted to limit the indoor drift of particulate matter into the terminal interiors.

The results of the second phase of our study on the impact of designated smoking location on PM2.5 concentrations were unsurprising. Our findings could largely be reduced to the observation that the further away one is from these locations, the less exposure to particulate matter results therefrom. Because the majority of pedestrian traffic at airport terminals likely occurs near the entrance and exit points, the placement of smoking venues close to these points likely results in greater risk to travelers. Furthermore, the correlation between cigarette count and PM2.5 (*R* ^2^ = 0.66) was very consistent with the results of an earlier study in the restaurant setting (*R* ^2^ = 0.64) [28].

What was perhaps unexpected, however, was our observation that PM2.5 correlated poorly with vehicle traffic passing through the terminal roads. We assessed this parameter because PM2.5, although an important marker of environmental air pollution [29], can be nonspecific as a surrogate for SHS in settings with multiple pollutants [2, 30], and studies have suggested that the vehicular contribution to urban ambient PM2.5 levels can reach the range of 32–40% [31, 32]. One study of tobacco exposure in airports in the United States and Europe explicitly declined to measure PM2.5 levels, citing this lack of specificity, and instead measured air nicotine levels [33]. However, when compared directly in controlled environments, a single cigarette produced over twice as much particulate emissions as two heavy-duty diesel trucks [34], and three cigarettes produced PM levels ten times greater than a single 2.0 litre diesel engine [35]. Therefore, it was possible that the PM2.5 contribution from vehicular emissions could simply have been muffled by the much greater contribution from tobacco smoke. Finally, our assessment of the PM2.5 burden from traffic by the absolute number of vehicles passing through a fixed point was a rather crude index. We did not take into account vehicle size, type of engine, or the presence of idling vehicles. All of these factors could have contributed to the lack of correlation between observed vehicle number and measured particulate emissions.

Other than measurements at randomly selected seating areas far into the terminal interiors as controls, all of our surveys were performed at terminal arrival areas which were partially enclosed by overhead departures above as well as terminal walls to one side. In a sense, arrival areas can be thought of as partially underground in many airports with such a layout. However, we were unable to directly assess the impact that these architectural elements may have on the possible promotion of microclimates which stagnate particulate matter from SHS and vehicular exhaust. We had initially attempted to compare simultaneous PM2.5 levels at departure areas above ground and arrival areas below but adjusting for large variances in cigarette number and traffic density proved to be too difficult. A microclimate effect caused by high buildings bounding narrow pedestrian streets has previously been described in an urban SHS study [16], but similar to the present investigation, the authors only measured particulate matter within the proposed microclimate without an open-air comparator. Another study in the restaurant setting found that overhead coverage outdoors indeed increased average SHS exposure by around 50% [36]. Several studies have investigated the utilization of computational fluid dynamics using k-ε turbulence models and models for particle transport to describe particle pollution in partially enclosed environments with good agreement with empirical data [37, 38]; such approaches potentially may be of use in modeling SHS in the airport setting.

Mean PM2.5 measurements in our study ranged from 3.1 to 31.3 *µ*g/m^3^, with a mean outdoor PM2.5 level of 17.9 *µ*g/m^3^. This figure exceeds the annual standard set by the US Environmental Protection Agency (EPA) for fine particles (PM2.5), which is currently set at 12 μg/m^3^ [39]. Although levels as high as 35 μg/m^3^ are permissible over 24 hours, individual point measurements taken on a per-minute basis in our study have exceeded 200 *µ*g/m^3^. This places not only airport workers but casual travelers at risk. Worryingly, the absolute number of passengers being exposed to these levels appears to be increasing. The utilization of ground transportation services at airport terminals has risen sharply in recent years, mirroring a general rise in air passenger numbers. At Los Angeles International (LAX), the second busiest airport in the US by enplanements, ground transport activity has risen every year from 2015 to 2018 [40], with much of this attributable to the expanding usage of ride sharing services. At LAX, these services, also called transportation network companies (TNC), saw a 6-fold increase from January 2016 to August 2018. In similar fashion, the top 15 most popular destinations nationwide for one particular platform consist exclusively of airports [41]. These statistics highlight the increasing vulnerability and potential for exposure of the travel population and lend urgency to the need to implement measures aimed at SHS and particulate matter reduction in this setting.

Although the movement of smoking areas further away from pedestrian traffic may be an effective measure in the short term, lasting benefits to public health are best achieved through smoke-free legislation [42]. However, despite progress in the enactment of comprehensive indoor smoking bans in many localities, outdoor bans have lagged behind, with controversy surrounding both the legality [43] as well as the effectiveness [44–46] of such ordinances, even if the hazardous nature of outdoor SHS has now been fully established [47]. Nevertheless, it is being increasingly recognized that “outdoor smoking is an important area for advancing smoke-free policy” [33], and calls for total, premise-wide bans in the airport setting are gaining in traction. Indeed, Article 8 of the World Health Organization's Framework Convention on Tobacco Control (FCTC) demands “universal protection by ensuring that all indoor public places, all indoor workplaces, all public transport and possibly other (outdoor or quasi-outdoor) public places are free from exposure to second-hand tobacco smoke” [48]. A broad interpretation of “public transport” should extend not only to vehicles but to the premises in which they operate, thus including the entire airport setting.

Our study has several limitations. As mentioned previously, sample size was low and vehicular density was measured rather crudely. We also did not attempt to correct for relative humidity (RH). Under conditions of high humidity, water vapor condenses on aerosol particles, causing them to grow hygroscopically and increasing apparent particle size [49]. However, our measurements were not conducted under high RH, and therefore, our conclusions were unlikely to have differed. For phase I, we only sought to assess indoor drift by comparing relative levels at indoor locations where RH was generally <60%, and these levels are unlikely to have much effect upon readings [50]. Although phase II was conducted outdoors, our surveys were performed in the evenings where RH again was generally <60%. Although a correction factor exists for RH, it has not been utilized in other SHS studies conducted in outdoor settings [25, 36, 47, 51–53]. Furthermore, we did not assess the impact of other particulates which may represent sources of both indoor and outdoor pollution in this setting. For example, particulate matter less than 10.0 microns in diameter (PM10) has also been found in SHS [35] and been noted to be an important contributor to the health effects observed in urban environments [54–56], including excess mortality [57]. Airborne fungi, ubiquitous to both airports and aircraft [58], can have interesting and even paradoxical effects on allergic diseases [59] but was similarly unassessed. Finally, we note that the negative health effects of ultrafine particles (UFP, particles with aerodynamic diameter <100 nm) have increasingly been recognized in recent years [60]. The much smaller size of these particles allows them to penetrate deeply into the respiratory tract and even into the systemic circulation, leading to unique pathological effects upon tissues [61, 62]. Airports in particular are rich sources of UFP due to emissions from aircraft engines [63], and studies have been conducted to examine their effects on surrounding communities [64, 65]. In addition, there has been research on the burden of UFP on airport employees, particularly in those who work in close proximity to aircraft [66], but the impact on travelers in common areas is unknown. Of note, both cigarette smoke [25, 67] and automobiles [68] are also sources of UFP, and the overall effect of these particles, particularly in the context of microclimate conditions, must await further research.

## 5. Conclusion

Secondhand tobacco smoke exposure in the airport setting represents a significant public health hazard. Although indoor smoking rooms have been removed from the majority of US airports, smoking activity continues to occur directly outside of terminal entrances. Exposure risk may be magnified due to the architectural designs of many terminals which place arrival areas directly underneath departures, creating partially enclosed spaces where particulate matter can potentially stagnate. Although the movement of designated outdoor smoking areas further away from entry and exit points may mitigate exposure, legislative initiatives aimed at comprehensive prohibition may be necessary to fully protect both travelers and airport employees from the health risks of secondhand smoke.

## Figures and Tables

**Table 1:** Characteristics of airports included in this study.

Airport arrival areas	Outdoor smoking restrictions?	Smoking areas in arrivals	Arrivals enclosed by departures overhead?	Ventilation	Terminal doors	Cigarette smell	Compliance with nonsmoking signs
Airport A	Yes	At terminal ends and in between on sidewalk; within 9 m of terminal entrances	Yes	Yes	Two sets	Yes	Poor; cigarette butts on ground even in nonsmoking areas

Airport B	Yes	Over 20 m away from entrance in secluded areas	Yes	Yes	One set	No	Moderate

Airport C	Yes	At terminal ends only but within 9 m of entrances	Yes	Yes	One set	No	Generally yes

**Table 2:** Comparison of PM2.5 levels by terminal area and raw data.

Terminal	Outdoor PM2.5 mean (*µ*g/m^3^)	Indoor PM2.5 mean (*µ*g/m^3^)	Seating PM2.5 mean (*µ*g/m^3^)	
Terminal 1	16.3	10.1	7.7	
Terminal 2	18.5	9.2	8.5	
Terminal 3	31.3	13	11.8	
Terminal 4	20.7	4.6	3.1	
Terminal 5	18.4	10.5	12.2	
Terminal 6	18	8.3	10.8	
Terminal 7	16.8	10.1	9.6	
Terminal 8	9.4	7.3	6.4	
Terminal 9	11.8	5.8	6.1	
Total mean (SD)	17.9 (6.1)	8.8 (2.6)	8.5 (3.0)	(*p* < *0.001*)

**Table 3:** Results of multiple linear regression analysis of cigarette and vehicle counts on PM2.5 measurements by location of designated smoking areas. Terminal 4 featured designated smoking areas immediately outside pedestrian entrances, while smoking areas at terminal 5 were located over 20 meters across the terminal road. *β* is the standardized beta coefficient.

	*β*	95% CI lower	95% CI upper	*R* ^2^	*p*
Terminal 4				0.70	<**0.001**
Lit cigarettes/min	0.83	0.55	1.11	0.66	<**0.001**
Vehicles/min	0.20	−0.08	0.49	0.01	0.15

Terminal 5				0.26	0.08
Lit cigarettes/min	0.36	−0.08	0.80	0.14	0.1
Vehicles/min	0.35	−0.09	0.79	0.13	0.1

## Data Availability

The data used to support the findings of this study are included within the article and Supplementary Materials file.

## Abbreviations

ANOVA: Analysis of variance
CDC: Centers for Disease Control and Prevention
CI: Confidence interval
FAA: US Federal Aviation Administration
MW: Mann–Whitney *U* test
PM: Particulate matter
SD: Standard deviation
SHS: Secondhand smoke
TNC: Transportation network company
UFP: Ultrafine particle.

## Supplementary Materials

Supplementary Materials Supplementary Materials S1: PM2.5 data by terminal location (.csv).
